# Immunogenicity of Self-Associated Aggregates and Chemically Cross-Linked Conjugates of the 42 kDa *Plasmodium falciparum* Merozoite Surface Protein-1

**DOI:** 10.1371/journal.pone.0036996

**Published:** 2012-06-04

**Authors:** Feng Qian, Karine Reiter, Yanling Zhang, Richard L. Shimp, Vu Nguyen, Joan A. Aebig, Kelly M. Rausch, Daming Zhu, Lynn Lambert, Gregory E. D. Mullen, Laura B. Martin, Carole A. Long, Louis H. Miller, David L. Narum

**Affiliations:** 1 Laboratory of Malaria Immunology and Vaccinology, National Institute of Allergy and Infectious Diseases, National Institutes of Health, Rockville, Maryland, United States of America; 2 Department of Rheumatology and Immunology, Changzheng Hospital, Second Military Medical University, Shanghai, People's Republic of China; 3 Laboratory of Malaria and Vector Research, National Institute of Allergy and Infectious Diseases, National Institutes of Health, Rockville, Maryland, United States of America; 4 Division of Imaging Sciences, School of Medicine, King’s College London, London, United Kingdom; 5 Novartis Vaccines Institute for Global Health S.r.l. (NVGH), Siena, Italy; London School of Hygiene and Tropical Medicine, United Kingdom

## Abstract

Self-associated protein aggregates or cross-linked protein conjugates are, in general, more immunogenic than oligomeric or monomeric forms. In particular, the immunogenicity in mice of a recombinant malaria transmission blocking vaccine candidate, the ookinete specific *Plasmodium falciparum* 25 kDa protein (Pfs25), was increased more than 1000-fold when evaluated as a chemical cross-linked protein-protein conjugate as compared to a formulated monomer. Whether alternative approaches using protein complexes improve the immunogenicity of other recombinant malaria vaccine candidates is worth assessing. In this work, the immunogenicity of the recombinant 42 kDa processed form of the *P. falciparum* merozoite surface protein 1 (MSP1_42_) was evaluated as a self-associated, non-covalent aggregate and as a chemical cross-linked protein-protein conjugate to ExoProtein A, which is a recombinant detoxified form of *Pseudomonas aeruginosa* exotoxin A. MSP1_42_ conjugates were prepared and characterized biochemically and biophysically to determine their molar mass in solution and stoichiometry, when relevant. The immunogenicity of the MSP1_42_ self-associated aggregates, cross-linked chemical conjugates and monomers were compared in BALB/c mice after adsorption to aluminum hydroxide adjuvant, and in one instance in association with the TLR9 agonist CPG7909 with an aluminum hydroxide formulation. Antibody titers were assessed by ELISA. Unlike observations made for Pfs25, no significant enhancement in MSP1_42_ specific antibody titers was observed for any conjugate as compared to the formulated monomer or dimer, except for the addition of the TLR9 agonist CPG7909. Clearly, enhancing the immunogenicity of a recombinant protein vaccine candidate by the formation of protein complexes must be established on an empirical basis.

## Introduction

Chemical conjugation is widely used to make haptens such as peptides and polysaccharides immunogenic. This is particularly significant for the development of several important human vaccines against polysaccharide moieties such as *Hemophilus influenzae* type b, *Streptococcus pneumoniae*, *Neisseria meningitidis* and *Salmonella enterica* serovar Typhi [1], [2], [3], [4]. Chemical conjugation can also be used on some proteins that are poor immunogens in order to enhance their immunogenicity. Conjugation effectively enhance the immunogenicity of the *Plasmodium falciparum* Pfs25, a transmission blocking malaria vaccine candidate, when recombinant Pfs25 was conjugated either to carrier proteins such as the outer-membrane protein complex of *Neisseria meningitidis* or ExoProtein A of *Pseudomonas aeruginosa*, (a detoxified form of exotoxin A from *P. aeruginosa*) or to itself (self-conjugation) [5], [6], [7]. Significant enhancement of Pfs25-specific antibody responses induced in both mice and rhesus monkeys was achieved. Similar results were observed when two other malaria antigens of *P. falciparum*, Pfs28 and AMA1, were conjugated to the ExoProtein A [7], [8]. In addition to enhancing immunogenicity, conjugation may overcome the restriction of host genetic backgrounds of vaccinees. While the single MSP1_19_ of *P. yoelii* failed to induce specific antibody responses in mice expressing H-2^s^ major histocompatibility complex haplotype, its conjugate coupled to diphtheria toxoid induced functional antibody responses in these mice [9].

The *P. falciparum* merozoite surface protein 1 (MSP1) is considered an important candidate for a vaccine approach targeting clinical disease or more specifically erythrocytic stage parasites. MSP1 is synthesized during blood stage development as a precursor with a molecular mass of ∼ 200 kDa, and later undergoes post-translational proteolytic processing. The proteolytic processing produces a C-terminal 42 kDa fragment (MSP1_42_) which is subsequently processed to 33 kDa and 19 kDa [10], [11]. Although inhibitory antibodies of MSP1_42_ are principally directed toward the 19 kDa fragment [12], the T cell epitopes on the 33 kDa MSP1 fragment enhance the immunogenicity and protective efficacy of the recombinant MSP1_42_ in non-human primates [13] and humans [14].

Several formulated MSP1_42_-based recombinant proteins of *P*. *falciparum* have been tested in *Aotus* monkeys [13], [15], [16], [17], [18] as well as in humans [19], [20], [21]. Protection against a lethal parasite challenge in *Aotus* monkeys has been reported, and is generally associated with a high level of MSP1_42_ specific antibody titers using Freund’s adjuvant [17], [18]. In contrast, in a phase 1 human trial a recombinant MSP1_42_/Alhydrogel™ vaccine formulation induced only a weak antigen-specific antibody response [19]. Various efforts have been made to enhance the immunogenicity and/or improve the efficacy of MSP1_42_–based vaccines, including the addition of toll–like receptor (TLR) agonists to the formulations [20], [21], [22], and the construction of chimeric proteins that replace the MSP1_33_ fragment either with other malarial antigens [23], [24] or adjuvanting protein fragments [25], [26], [27], [28]. Only the use of TLR agonists in vaccine formulations has subsequently been evaluated in human clinical trials, showing enhanced antibody responses [20], [21].

In this study, we evaluated whether the immunogenicity of MSP1_42_ in mice is enhanced when presented as 1) a self-associated aggregated protein or 2) chemically conjugated to a carrier protein formulated on Alhydrogel with or without CPG 7909, a synthetic B type CpG-ODN (unmethylated oligodeoxynucleotide containing cytosine-guanosine (CpG) dinucleotide motifs). In contrast to our previously reported findings for Pfs25 [5], [6], [7], neither self-association nor chemical conjugation to ExoProtein A (EPA) enhanced the immunogenicity of recombinant MSP1_42_ in mice.

## Materials and Methods

### Ethics Statement

Rodent studies were carried out in compliance with the National Institutes of Health guidelines and an animal care and use committee-approved protocol.

### MSP1_42_ Antigens and Carrier Protein

MSP1_42_-FUP and MSP1_42_-FVO are two allelic forms of recombinant *P. falciparum* MSP1_42_, with an E-KNG or Q-KNG MSP1_19_ phenotype, respectively. The recombinant MSP1_42_ proteins were expressed in *Escherichia coli*, refolded, purified and characterized as previously described [16], [29]. The MSP1_33_ fragment of MSP1_42_-FUP contains a single unpaired cysteine residue, which is absent in the MSP1_33_ fragment of the FVO allele that provided an unpaired sulfhydryl group for the conjugation of the MSP1_42_-FUP to a carrier protein modified by maleimide groups (see below). The aggregated MSP1_42_-FVO protein was produced following the reported purification process, except the S30 reverse-phase chromatography step was replaced with a hydrophobic interaction chromatography (HIC) step following the refold by rapid dilution. The HIC step used a Phenyl 650 M (Tosoh Biosciences, Montgomeryville, PA) column equilibrated in 50 mM Tris-HCl, pH 7.4, 1 mM EDTA, 1.2 M NaCl, 1 M Urea at 200 cm/h. Sodium chloride crystals were added to the rapid dilution refolded protein to make a 1.2 M final concentration and loaded onto the Phenyl 650 M at 200 cm/h after which unbound proteins were washed from the column using equilibration solution. The MSP1_42_-FVO protein eluted from the column with 20 mM Tris-HCl, pH 7.4, 1 mM EDTA, 1 M Urea was pooled and polished using a Superdex 200 size exclusion column (GE Healthcare, Piscataway, NJ) equilibrated with PBS with 0.02% polysorbate 80, pH 7.4. The EPA used as a carrier protein in this study, was produced in an *E. coli* expression system, as previously described [7].

### Maleimide Modification

Two chemical linkers, *N*-[ε-maleimidocaproyloxy]sulfosuccinimide ester (Sulfo-EMCS) and succinimidyl-[(*N*-maleimidopropionamido)-diethyleneglycol] ester (NHS-PEO_2_-Maleimide) (Pierce Inc., Rockford, IL), containing hydrocarbon and polyethylene glycol spacers, respectively, were used to modify EPA. These two chemical linkers are heterobifunctional cross-linkers with an N-hydroxysuccinimide (NHS) ester and a maleimide group at each of their termini. The maleimide-reaction pH was fixed at 7.2 and the EPA concentration was fixed at 2 mg/mL. Three other parameters: reaction time, reaction temperature and chemical linker concentration were optimized by employing a model using a three-level Box-Behnken design and the JMP statistical software (SAS Institute, Inc., Cary, NC). The final parameters used for these conditions were a reaction time of 60 min, a reaction temperature at 22°C and linker concentration of 2 mM.

The EPA was buffer exchanged to PBS-E (1× PBS, 5 mM EDTA, pH 7.2) using 5 kDa MWCO spin filter (Millipore, Billerica, MA). The sulfo-EMCS dissolved in PBS-E and NHS-PEO_2_-Maleimide dissolved in Dimethylsulfoxide were added to the EPA, respectively. The mixtures were incubated under the defined conditions with gentle shaking. At the end of the reaction, stop solution (1 M Tris-HCl pH 7.4) was added to a final concentration of 20 mM and then the buffer was immediately exchanged to PBS-E. The maleimide modified EPA (maleimide-EPA) was characterized by reversed-phase HPLC and by maleimide measurement.

### Conjugation

The purified MSP1_42_-FUP protein was reprocessed prior to conjugation using a preparative SEC column (16×60 mm, Superdex 200) equilibrated with PBS-EU (1×PBS, 1 mM EDTA, 5 M urea, pH 7.2) in order to remove the 0.2% polysorbate 80 present in the protein solution and expose the single unpaired cysteine residue on the MSP1_33_ fragment for conjugation. The conjugation conditions were 22°C for 1 hour with gentle shaking in PBS-EU. MSP1_42_-FUP and EPA-maleimide were mixed based on an equal number of moles of free sulfhydryl and maleimide groups. The MSP1_42_-FUP-EPA conjugate was loaded on a SEC column (16/60 Superdex 200) equilibrated with PBS-A (1×PBS, 0.5 M arginine, pH 7.2). The peak elution fractions containing MSP1_42_-FUP-EPA conjugates were selected and pooled based on a Coomassie blue stained SDS-PAGE gel.

### Characterization of Recombinant Protein Intermediates and Conjugates

#### A. SDS-PAGE and Western blotting

The EPA conjugates of MSP1_42_-FUP were characterized by Coomassie blue stained SDS-PAGE under non-reduced condition [29]. Western-blots were performed as described previously using antigen specific monoclonal antibodies AD223 and 13E3-53 [30].

#### B. Maleimide measurement

Maleimide groups were measured using Ellman’s reaction (indirect) as per manufacturer’s instructions (Pierce Inc., Rockford, IL). Briefly, the maleimide-EPA samples were titrated with the solution of Cysteine Hydrochloride Monohydrate. After the addition of Ellman’s reagent to each reaction, the absorbance was read at 405 nm. Based on the cysteine consumed in the reaction, the concentration of maleimide in the maleimide-EPA sample was determined relative to the standard curve of the cysteine with increasing concentrations. The number of maleimide groups added onto the EPA was obtained by dividing the moles of maleimide by the moles of EPA.

#### C. Composition analysis of MSP1_42_-FUP-EPA conjugate by amino acid analysis

Amino acid analysis was performed by the W.M. Keck Facility at Yale University. These results were used to calculate the molar ratios of MSP1_42_-FUP to EPA (average conjugation ratio) [31] and the concentrations of MSP1_42_-FUP molecules.

#### D. Reversed Phase-HPLC

Recombinant EPA was characterized, pre- and post-modification with maleimide groups, on an analytical 2.1×250 mm C4 column (GraceVydac, Hesperia CA) connected to a Waters 2695 HPLC system (Waters, Milford, MA). The column was equilibrated in 95% acetonitrile +0.1% TFA (trifluoroacetic acid) and the proteins eluted in a gradient of 40% to 58% acetonitrile +0.1% TFA over 36 minutes at a flow rate of 0.2 mL/minute.

#### E. SEC-MALS-HPLC

SEC-MALS-HPLC [7], [29] was performed on a Waters 2695 HPLC system, with an in-line Wyatt Dawn EOS light scattering detector, a quasi-elastic light scattering detector (QELS) and an Optilab refractive index detector (Santa Barbara, CA). The MSP1_42_-FVO self-associated aggregates or MSP1_42_-FUP-EPA conjugate were analyzed on a G4000SWxl size exclusion column (Tosoh Biosciences, Montgomeryville, PA) equilibrated with the following solution: 1.04 mM KH_2_PO_4_, 2.97 mM Na_2_HPO_4_, 308 mM NaCl, and 0.02% azide, pH 7.4 at a flow rate of 0.5 mL/min.

#### F. Endotoxin level measurement

Endotoxin levels of the various antigens were measured using *Limulus* amoebocyte lysate in a 96-well plate with chromogenic reagents and PyroSoft software (Associates of Cape Cod Inc., East Falmouth, MA) before administration. The endotoxin values were all less than 41 EU/mg of recombinant MSP1_42_.

### Animal Studies and Serological Assays

Antigens were formulated on 1600 µg/mL Alhydrogel (Brenntag Biosector, Denmark) with or without the addition of 20 µg/dose CPG 7909 (Coley Pharmaceutical Group, Wellesley, MA). For the formulation containing CPG 7909, the antigens were first formulated on Alhydrogel followed by the addition of CPG 7909. The adsorption of the antigens or CPG 7909 to Alhydrogel was examined by silver stained SDS-PAGE [32], [33]. BALB/c mice (Charles River Laboratories, Frederick, MD) were used and randomly assigned into experimental groups each containing 10 mice. The mice were immunized intramuscularly two times on days 0 and 28 with the delivery volume of 50 µL for each immunization. Mouse sera were collected two weeks after the second immunization (on day 42 in the first study) or two, four and six weeks (on days 42, 56 and 70 in the second study) after the second immunization for assessment of antigen specific antibody titers by ELISA (see below).

Two independent mouse studies were carried out in compliance with the National Institutes of Health guidelines and an animal care and use committee-approved protocol. In the first study, the antibody levels induced by the Alhydrogel formulations of MSP1_42_-FVO dimer and MSP1_42_-FVO aggregate were compared at the doses of 1, 3 and 10 µg. In the second study, the antibody levels induced by the Alhydrogel formulations or Alhydrogel plus CPG 7909 formulations of MSP1_42_-FUP monomer and two types of MSP1_42_-FUP-EPA conjugates were compared at the doses of 5 and 15 µg.

Enzyme-linked immunosorbent assay (ELISA) was performed on each individual mouse serum to measure the antibody titer following a standardized protocol [32], [34]. A Mann-Whitney U Test was performed in the first mouse study to test for significant differences of antibody titers between the two groups receiving different kinds of antigens at each dose level. If the *P* value was less than 0.05, the differences were considered significant. In the second study, Kruskal-Wallis One-Way ANOVA was performed among the groups at each dose level on any of three sera collection days. If the *P* value was less than 0.025, a post hoc analysis of Student-Newman-Keuls was performed. If the *P* value of Student-Newman-Keuls was less than 0.05, the differences were considered significant. For the IgG subclass analysis, ELISA was performed on the pooled sera of each group, which were pooled based on the equivalent ELISA unit of each individual serum [32], [35].

## Results

### Preparation and Characterization of MSP1_42_-FVO Self-associated Aggregate and MSP1_42_-FUP-EPA Conjugates

**Figure 1:**
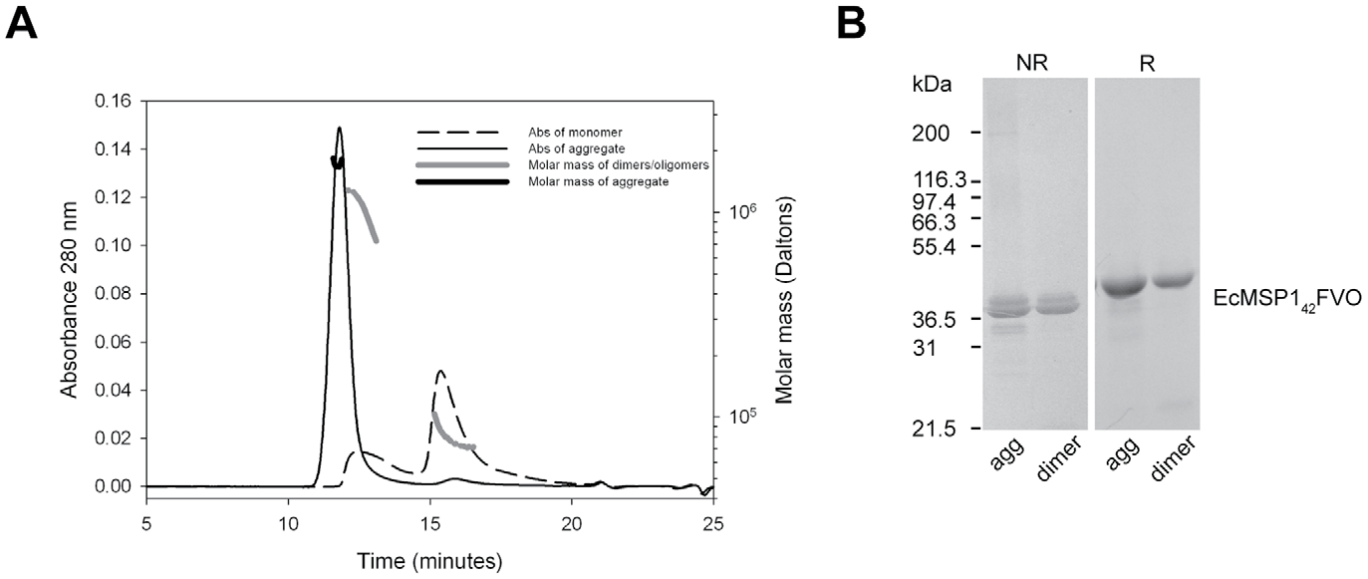
SEC-HPLC-MALS and Coomassie blue stained SDS-PAGE gel analysis of aggregated MSP1_42_-FVO. (A) Size exclusion chromatography with multi-angle light scattering and (B) SDS-PAGE analysis run on a 4–20% gradient Tris–glycine polyacrylamide gel under non-reducing (NR) and reducing (R) conditions.

A MSP1_42_–FVO self-associated aggregate was produced while developing a modified purification process that aimed to replace usage of a Source 30 pilot-scale reversed phase column with a hydrophobic interaction column in order to avoid the use of organic solutions during pilot-scale manufacturing. The molar mass of the MSP1_42_-FVO aggregate in an aqueous solution was determined by SEC-MALS-HPLC to be approximately 1.7 MDa for 96% of the total peak area as compared to a purified form following a standard procedure, which yielded 72% dimers and 28% multimers with a molar mass of approximately 80–100 kDa and 0.700–1MDa, respectively (Figure 1A). Analysis of the same protein lots by Coomassie blue stained SDS-PAGE analysis under reduced and non-reduced conditions showed that a predominant band at approximately 42 kDa was observed, which is consistent with the expected mass of 42,173 Da. Based on protein mobility in the presence of SDS, the solution state of the self-associated aggregate appeared to be primarily due to hydrophobic or ionic interactions, and not due to disulfide bond formation between different forms of MSP1_42_-FVO (Fig. 1B).

**Figure 2:**
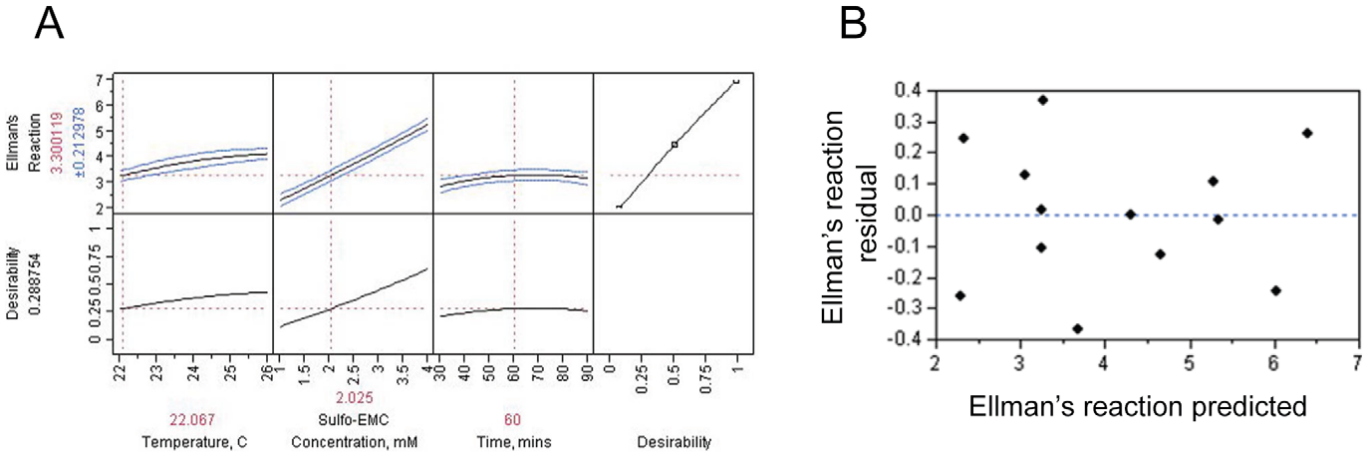
Multi-parameter analysis of linker substitution. Response curves for the analysis of temperature, modifier concentration and time on the linker substitution of EPA using a Box-Behnken model (A). Residual plot of the Ellmans’ reaction showing there is no systematic pattern (B). A linear regression model was used for analysis given the following assumptions: relationships between dependent and independent variables are linear, no serial correlation, the response variables are normally distributed, and have the same variance.

To determine the best conditions for linker addition, a Surface Response Mode (RSM) was used to pinpoint the maximum or minimum condition for three factors considered key for process development: temperature, pH and linker concentration. To evaluate these variables together, a Box-Behnken’s RSM was used to determine the optimum condition for the maleimide modification reaction based on the following conditions: temperature (22–26°C), Sulfo-EMC concentration (1.0–4.0 mM) and reaction time (30–90 minutes). All three factors were statistically significant, p<0.05, and played an important role in the number of modifications observed based on the Ellman’s Reaction. However, due to process development constraints, and tolerability of temperature and pH to marked linker substitution, the following conditions were used: reaction temperature 22°C, linker concentration of 2 mM, and reaction time 60 min (Figure 2A). To test the validity of the Box-Behnken’s RSM, the residual plot was investigated. Figure 2B shows the Ellman’s reaction was randomly dispersed around the horizontal axis with no trend, which is consistent of a linear regression model. Furthermore, the fitted model accounted for (R-squared) 96% of the variation in Ellman’s reaction. We were interested in only main factors in the RSM and did not account for squared terms, interactions or synergistic effects. Further process optimization, using the Box-Behnken RSM, could address higher order terms, and surface response model curvatures.

**Figure 3:**
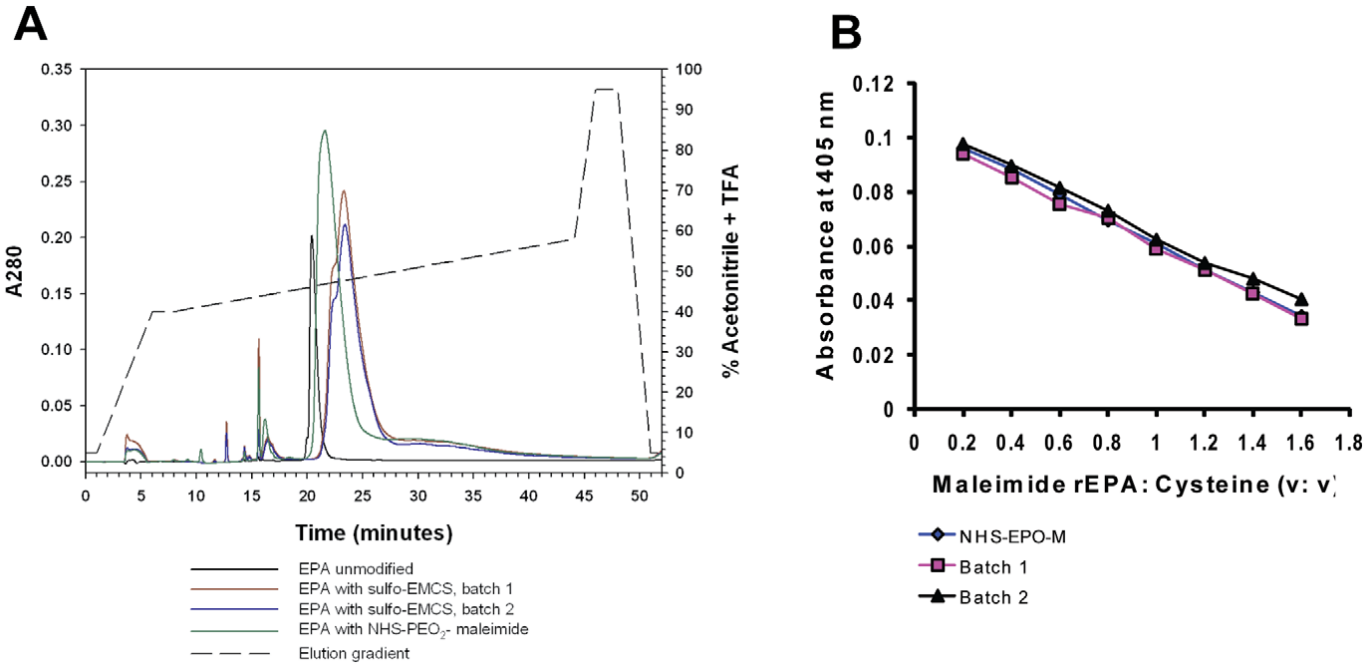
Characterization of chemically modified EPA. Analysis of un-modified EPA, EPA_APA_ batch 1, EPA_APA_ batch 2 and EPA_PEO_ by RP-HPLC (A), and titration analysis of EPA_APA_ batch 1, EPA_APA_ batch 2 and EPA_PEO_ (B) are shown.

Batches of chemical cross-linked MSP1_42_-FUP-EPA conjugates were prepared using two different maleimide cross-linkers Sulfo-EMCS and NHS-PEO_2_-maleimide. The composition of each linker indicates that the solubility properties may be different, even though this did not appear to impact the solubility of the conjugates (data not shown). Recombinant EPA_APA_ and EPA_PEO_, modified by Sulfo-EMCS and NHS-PEO_2_-maleimide, respectively, were prepared and the addition of the bifunctional linkers was monitored by RP-HPLC analysis. The retention times as well as the peak shape of the modified EPA shifted with the addition of the maleimide groups onto the protein (Figure 3A). The extent of the shift in retention time appeared dependent on the number and physical properties of the chemical modifiers (Figure 3A and data not shown). The number of maleimide groups added on EPA was assessed by titrating with cysteine and measuring the absorbance at 405 nm (Figure 3B). The titration curves of two batches of EPA_APA_ and one batch of EPA_PEO_ were similar (Figure 3B). Based on the titration curves and the standard curve of cysteine, the number of maleimide groups added onto the EPA was calculated to be 5.0 and 4.7 for the two batches of EPA_APA_ and 5.0 for the EPA_PEO_.

**Figure 4:**
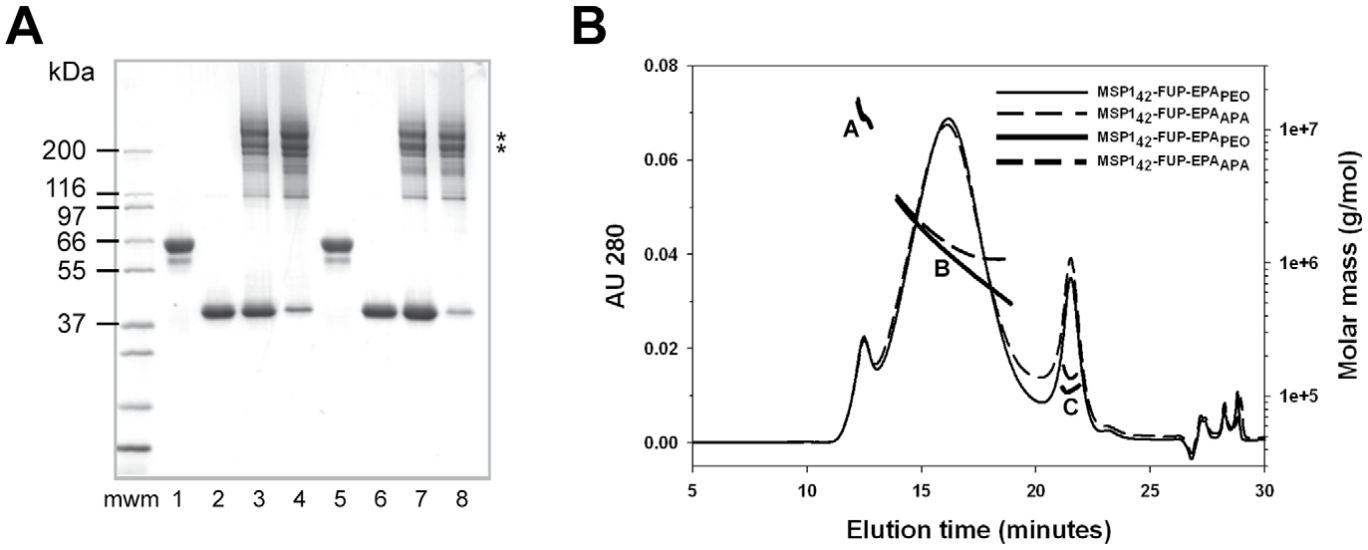
Characterization of MSP1_42_-FUP-EPA conjugates. Panel (A) Coomassie blue stained SDS-PAGE gel analysis of maleimide-EPA (lanes 1 and 5); monomeric MSP1_42_-FUP (lanes 2 and 6), un-purified conjugation mixture of MSP1_42_-FUP-EPA_APA_ (lane 3), bulk purified MSP1_42_-FUP-EPA_APA_ (lane 4); un-purified conjugation mixture of MSP1_42_-FUP-EPA_PEO_ (lane 7) and bulk purified MSP1_42_-FUP-EPA_PEO_ (lane 8). The asterisks indicate the conjugates with 3∶1 and 4∶1 ratio. Panel (B) SEC-HPLC-MALS analysis. Solid and dashed lines represent absorbance at 280 nm and molecular mass for MSP1_42_-FUP-EPA_PEO_ and MSP1_42_-FUP-EPA_APA_, respectively. The capital letters A, B and C indicate the profile peaks of each conjugate.

**Table 1:** Anti-MSP1_42_-FVO antibody titers in mice.

	Anti-MSP1_42_-FVO units^a^ (Geometric mean ± SEM)		
	Dose 1 µg	Dose 3 µg	Dose 10 µg
MSP1_42_-FVO dimer	715±2890	6297±4940	23667±5022
MSP1_42_-FVO aggregate	2444±4950	4973±8357	57936±7457

a The antibody titers were compared at any of three doses levels with Mann-Whitney U Test. No significant differences were presented as the *P* values were all more than 0.05.

**Table 2:** Anti-MSP1_42_-FUP antibody titers in mice.

	Anti-MSP1_42_-FUP units (Geometric mean ± SEM)^a^					
	Dose 5 µg			Dose 15 µg		
	Day 42	Day 56	Day 70	Day 42	Day 56	Day 70
MSP1_42_-FUP monomer	1309±2109	1570±2538	1241±1882	7588±4477	9493±4050	8369±4306
MSP1_42_-FUP-EPA_APA_	1770±2701	2102±3281	1827±2369	5685±2116	5627±2630	4275±2335
MSP1_42_-FUP-EPA_PEO_	1009±1716	1288±1665	1062±2023	8064±3029	7744±3732	4881±1692
MSP1_42_-FUP monomer CPG 7909	109075±21780	89277±20008	44237±4929	107850±13162	118401±13938	52794±5243
MSP1_42_-FUP-EPA_APA_ CPG 7909	54327±10650	45206±9532	21341±5183	103919±17292	59705±8829	33042±4018
MSP1_42_-FUP-EPA_PEO_ CPG 7909	73995±15475	33363±10701	24742±6113	108278±15234	75767±11789	34766±3467

a The antibody titers were compared at two dose levels on any of three serum collection days with Kruskal-Wallis One-Way ANOVA. As the *P* values were all less than 0.025, the post hoc analysis of Student-Newman-Keuls was performed. The differences between the groups of conjugated and unconjugated immunogens were not significant (MSP1_42_-FUP-EPA_APA_ or MSP1_42_-FUP-EPA_PEO_ vs. MSP1_42_-FUP monomer and MSP1_42_-FUP-EPA_APA_ CPG 7909 or MSP1_42_-FUP-EPA_PEO_ CPG 7909 vs. MSP1_42_-FUP monomer CPG 7909) (*P*>0.05), whereas the differences between the groups with and without CPG 7909 were significant (MSP1_42_-FUP monomer CPG 7909 vs. MSP1_42_-FUP monomer, MSP1_42_-FUP-EPA_APA_ CPG 7909 vs. MSP1_42_-FUP-EPA_APA_ and MSP1_42_-FUP-EPA_PEO._

The EPA_APA_ and EPA_PEO_ carriers were conjugated with MSP1_42_-FUP and the resultant conjugates were purified by preparative SEC. The SEC elution fractions were analyzed by Coomassie blue stained SDS-PAGE and relevant fractions were pooled (Figure 4A). Analysis of the mobility of the conjugates by Coomassie blue stained SDS-PAGE indicated that the conjugation products were chemically cross-linked complex protein-protein mixtures with ratios ranging from 1∶1 MSP1_42_-FUP per carrier to approximately 6∶1. The predominant conjugated forms appeared at a ratio of 3∶1 and 4∶1 (see Figure 4A asterisks). A minor quantity of unreacted MSP1_42_-FUP was observed in the SEC pool. The average conjugation ratio for MSP1_42_-FUP-EPA_APA_ and MSP1_42_-FUP-EPA_PEO_ by amino acid analysis was 3.9 and 3.8, respectively. Thus the results obtained by mobility on SDS-PAGE and amino acid analysis were consistent for the ratio of MSP1_42_-FUP and EPA by mass ratios and molar ratios. Considering that the unreacted MSP1_42_-FUP was not completely removed from either conjugate, the average conjugation ratios should be slightly lower than the reported values. Analysis by SEC- MALS-HPLC showed the presence of three populations or peaks for each conjugate with the second peak representing the major conjugated form with the major peak consisting of 80% of total protein for the MSP1_42_-FUP-EPA_APA_ and 81% of the total protein for the MSP1_42_-FUP-EPA_PEO_ (Figure 4B). The weighted average masses of the MSP1_42_-FUP-EPA_APA_ were 1.1 × 10^7^, 1.6 × 10^6^ and 1.4 × 10^5^ Da, respectively, whereas the weighted average masses for the MSP1_42_-FUP-EPA_PEO_ of three peaks were 1.1×10^7^, 1.3×10^6^ and 1.1×10^5^ Da (Figure 4B, A–C, respectively). Since the predominant weighted average mass for each conjugate by SEC-MALS-HPLC was greater than the ratios determined by Coomassie blue stained SDS-PAGE, this indicates that a non-covalent association exists in the presence of the 0.5 M arginine, which was used to stabilize the solubility of the conjugates. The conjugates in this form were stable to freeze-thaw by analytical SEC-MALS-HPLC and were filterable through a 0.22 µm filter (data not shown). Two conformation-dependent monoclonal antibodies, AD223 and 13E3-53 [30], were used to assess the structural integrity of the conjugated MSP1_42_-FUP proteins by Western blot analysis. Each protein band observed by Coomassie blue stained SDS-PAGE appeared to be recognized by both monoclonal antibodies (data not shown), demonstrating that the conformational structure of the 19 kDa fragment of MSP1_42_-FUP remained intact during the process of conjugation.

### Assessment of Immunogenicity of Aggregated, Chemically Conjugated or Dimeric Recombinant MSP1_42_

To evaluate whether the MSP1_42_-FVO self-associated aggregate or MSP1_42_-FUP-EPA conjugates could enhance antigen specific antibody responses compared to the predominately monomeric or dimeric forms, two mouse studies were performed with the immunogens formulated on Alhydrogel, and in the case of the MSP1_42_-FUP-EPA conjugate with or without CPG 7909. The antibody titers of each mouse serum were measured by ELISA. In the first study, the antibody titers induced by aggregated or dimeric MSP1_42_-FVO formulated on Alhydrogel were compared at the doses of 1, 3 and 10 µg. No significant differences were observed at any of these dose levels although there is a trend of a higher response for the aggregated form of MSP1_42_-FVO compared to the dimer (Table 1). In the second study, the antibody titers induced by the two different MSP1_42_-FUP-EPA conjugates and unconjugated MSP1_42_-FUP formulated on Alhydrogel with or without CPG 7909 were compared at the doses of 5 and 15 µg. When the comparison was performed between the conjugated and unconjugated immunogens, again no significant differences were observed at these dose levels on any of three serum collection days (Table 2). However, at the 5 µg dose, the MSP1_42_-FUP-EPA_APA_ conjugate consistently had a higher response when formulated without CPG 7909 and the MSP1_42_-FUP monomer consistently had a higher response when formulated with CPG 7909. When the comparison was performed between formulation with and without CPG 7909, significant differences were observed. Antibody levels induced by the formulations with CPG 7909 were significantly higher than those induced by the formulations without CPG 7909 at both doses of 5 and 15 µg on all three serum collection days (Table 2). On day 42, the differences in antibody titers reached 31∼ 83-fold higher at the dose of 5 µg and 13∼ 18-fold higher at the dose of 15 µg.

**Table 3:** IgG subclass analysis of MSP1_42_-FUP-specific mouse antibodies elicited by the formulations with CPG 7909.

	IgG1 : IgG2a ratio					
	Dose 5 µg			Dose 15 µg		
	Day 42	Day 56	Day 70	Day 42	Day 56	Day 70
MSP1_42_-FUP, CPG 7909	1.28	1.23	1.26	1.04	1.01	1.11
MSP1_42_-FUP-EPA_APA_,CPG 7909	0.88	0.77	0.69	0.70	0.58	0.51
MSP1_42_-FUP-EPA_PEO_,CPG 7909	0.73	0.71	0.50	0.71	0.62	0.57

IgG subclass analysis was performed on the pooled sera of the mouse groups to characterize the type of antibody responses induced by the formulations with and without CPG 7909. The Alhydrogel formulations of both conjugated and unconjugated MSP1_42_-FUP without CPG 7909 predominately induced an IgG1 response (data not shown). With the addition of CPG 7909 to the Alhydrogel formulations, an IgG2a subclass level was greatly increased. Both IgG1 and IgG2a were the predominant IgG subclasses in these immune sera. The IgG1 to IgG2a ratios from day 42 to day 70 were different between the groups of conjugated and unconjugated MSP1_42_-FUP. The ratios decreased in the sera elicited by the conjugated MSP1_42_-FUP, indicating that in those sera the IgG1 waned more quickly than the IgG2a (Table 3).

## Discussion

An effective malaria vaccine is urgently needed to augment existing control measures for individuals living in malarial endemic areas. Unfortunately, several investigative malaria vaccines tested in phase 1 or 2 trials have not induced antibody levels that have warranted further development [19], [36], [37], [38]. Self-assembled virus like particles [39] including the leading malaria vaccine RTS,S that contains the circumsporozoite protein fused with the hepatitis B surface antigen are capable of inducing protective responses in humans. RTS,S has protected about 50% of vaccinees for a duration of 12–18 months in Phase 2 trials [40], [41], and in a recent Phase 3 trial [42]. In preclinical studies, a non-human primate immunogenicity study demonstrated that recombinant Pfs25H protein, a mimic of the sexual stage specific protein Pfs25, conjugated to the outer membrane complex of *N. meningitidis* increased antibody titers to a greater degree than their monomeric forms and increased the apparent duration of the antigen specific antibodies [5]. Similar observations of an increase in antibody titers have been made when recombinant Pfs25H was conjugated to a different carrier protein i.e., EPA or to itself [6], [7].

Unfortunately, based on the results reported here, whether recombinant MSP1_42_ was presented as a uniform non-covalently associated aggregate or chemically cross-linked to EPA no significant increase in antibody responses were observed in mice (Tables 1 and 2). The basis for the differences in antibody responses observed for recombinant Pfs25 conjugates and those described here is unclear. Both Pfs25 and MSP1_42_ native proteins contain 4 or 2 epidermal growth factor like domains, respectively [10], [43]. Native Pfs25 is believed to be deficient of T cell epitopes, while MSP1_42_ contains the 33 kDa fragment which is believed to already provide T cell help. The presence of the MSP1_33_ fragment may negate the benefit of chemical cross-linking to EPA.

CpG ODN has been demonstrated to be an effective adjuvant for vaccines against a variety of pathogens including malarial antigens [20], [32], [35], [44], [45], [46], [47], [48]. When CpG ODN was added to an aluminum hydroxide formulation, the combination of both adjuvants altered the subclass profile of the IgG response from a predominant IgG1 (Th2) to a more balanced IgG1 and IgG2a, reflecting both Th1 and Th2 patterns of immune responses and leading to an increased total antibody level [32], [35]. Moreover, the protective effect of the antibodies against the challenge of malaria parasites in mice was improved by the addition of CpG ODN to the Montanide formulation of *P. yoelii* MSP1_19_ [45]. While the aggregated and conjugated MSP1_42_ failed to enhance the antibody responses, the addition of CPG7909 to the Alhydrogel formulation of the MSP1_42_ significantly enhanced the specific antibody levels in mice with balanced IgG subclasses, which is consistent with the result observed in our previous phase 1 human trial [20].

In summary, neither recombinant MSP1_42_ self-associated aggregates nor chemical cross-linked conjugates enhanced the immunogenicity of the MSP1_42_ compared to monomeric or oligomeric forms of the antigen. The addition of CPG 7909 into the Alhydrogel formulation of the MSP1_42_-EPA conjugates significantly enhanced the specific antibody levels in mice as compared to the formulation using Alhydrogel as a single adjuvant. Even though the MSP1_42_-EPA conjugates failed to enhance MSP1_42_ immunogenicity, the conjugation procedure including the use of design of experiments may be used as a platform for development of other protein-protein conjugates through chemical modification of both antigen and carrier, or use of innate or genetically engineered free sulfhydryl groups for protein-protein coupling. Further investigation is required to understand the benefit of this protein-protein conjugation strategy for the development of investigational malaria vaccines or other vaccines.
